# How is neuromuscular fatigability affected by perceived fatigue and disability in people with multiple sclerosis?

**DOI:** 10.3389/fneur.2022.983643

**Published:** 2022-10-17

**Authors:** Nicolas Royer, Kyla Coates, Saied Jalal Aboodarda, Jean-Philippe Camdessanché, Guillaume Y. Millet

**Affiliations:** ^1^Inter-University Laboratory of Human Movement Biology, University of Lyon, UJM-Saint-Etienne, Saint-Etienne, France; ^2^School of Exercise and Health Sciences, Faculty of Health and Social Development, University of British Columbia Okanagan Kelowna, Kelowna, BC, Canada; ^3^Faculty of Kinesiology, University of Calgary, Calgary, AB, Canada; ^4^Department of Neurology, University Hospital of Saint-Etienne, Saint-Etienne, France; ^5^Institut Universitaire de France (IUF), Paris, France

**Keywords:** fatigue, fatigability, disability, multiple sclerosis, muscle weakness, corticospinal responses

## Abstract

Whereas fatigue is recognized to be the main complaint of patients with multiple sclerosis (PwMS), its etiology, and particularly the role of resistance to fatigability and its interplay with disability level, remains unclear. The purposes of this review were to (i) clarify the relationship between fatigue/disability and neuromuscular performance in PwMS and (ii) review the corticospinal and muscular mechanisms of voluntary muscle contraction that are altered by multiple sclerosis, and how they may be influenced by disability level or fatigue. Neuromuscular function at rest and during exercise are more susceptible to impairement, due to deficits in voluntary activation, when the disability is greater. Fatigue level is related to resistance to fatigability but not to neuromuscular function at rest. Neurophysiological parameters related to signal transmission such as central motor conduction time, motor evoked potentials amplitude and latency are affected by disability and fatigue levels but their relative role in the impaired production of torque remain unclear. Nonetheless, cortical reorganization represents the most likely explanation for the heightened fatigability during exercise for highly fatigued and/or disabled PwMS. Further research is needed to decipher how the fatigue and disability could influence fatigability for an ecological task, especially at the corticospinal level.

## Introduction

Multiple sclerosis (MS) is a chronic autoimmune disease involving demyelination degeneration in the central nervous system (CNS), typically in subcortical brain areas and their connections. However, in some cases of MS, cortical neuronal loss occurs only in the gray matter, without demyelination of cerebral white matter (1). Compromised action potential propagation and conduction velocity in people with MS (PwMS) results functional disability that is worsened as demyelination and lesion load increase with disease progression (2). The level of MS-related disability is typically quantified by the expanded disability status scale (EDSS) (3), characterized by the impairment of different functional neurological systems (i.e., cerebral: cognitive ability and memory, pyramidal: motor function, etc.) and the ability to walk on a scale from 0 to 10. In the literature, it is generally considered that a low disability level (typical EDSS: < 3) represents low impairment caused by the disease and preserved functional capacity (minimal handicap), whereas high disability (typical EDSS: >5) involves impaired functional capacity, including poor walking ability with a restrained walking perimeter and/or the patient requiring walking aids (4). There are three phenotypes defined for MS. Relapsing-remitting MS (RRMS) is the most common clinical course (85%) and is characterized by alternating periods of remission and recovery, while 15% never experience periods of recovery and are diagnosed with primary progressive MS (PPMS). The majority of people with RRMS eventually progress to a continually worsening condition called secondary progressive MS (SPMS) (5). Although disability in MS is heterogeneous and dependent on the location of demyelination or cortical lesions, the hallmark of the disease is motor dysfunction; including muscle weakness, spasticity, and motor fatigability (6).

Motor fatigability (simply called fatigability throughout this review) can be defined as a reduction in the capacity to produce maximal power output and/or maximal voluntary or evoked force after exercise (7–9). Though often overlooked compared to muscle weakness or confused with MS-related fatigue (see below), fatigability is a significant concern for people with MS, as a limited ability to sustain functional tasks is often reported (10, 11). For example, in the clinical setting, some patients report that walking can only be sustained for a limited amount of time before a rest is required to re-initiate movement again. Fatigability may occur independent of muscle weakness, as shown by Schwid et al. (12) who showed no correlation between baseline maximal voluntary torque output and fatigability induced by a 30-s isometric fatiguing task for both upper and lower limb muscles. It is assessed through objective neuromuscular and performance evaluations, whereby the underlying central (i.e., cortical and/or spinal circuitries) and peripheral (i.e., distal to the neuromuscular junction) mechanisms can be assessed (13, 14). Unfortunately, the majority of MS literature has failed to properly differentiate fatigability from the subjective sensation of fatigue experienced by PwMS, so much confusion exists about its etiology and its impact.

Not to be confused with fatigability, MS-related fatigue is one of the most common symptoms of MS. MS-related fatigue is reported by 45–78% of people across all clinical phenotypes, and is often described by PwMS as one of the most disabling symptoms experienced (15–18). MS-related fatigue is defined as a “*subjective sensation of weariness, an increasing sense of effort, a mismatch between effort expended and actual performance, or exhaustion*” and is measured with self-report scales (19). MS-related fatigue can be differentiated from the fatigue experienced by healthy individuals as it is not substantially improved by sleep or rest, and can be aggravated by heat, and mental or physical exertion (15, 20, 21). Most of the documented studies that have measured MS-related fatigue have used questionnaires such as the Fatigue Severity Scale (FSS) (22) and the Modified Impact Fatigue Scale (MFIS) (15, 20, 23). Based on these subjective questionnaires, cut offs were created to distinguish fatigued vs. non-fatigue PwMS (4 for FSS; 38 for MFIS) (23). The FSS focuses heavily on physical expressions of fatigue (i.e., fatigability) while the MFIS encompasses physical, cognitive and psychosocial dimensions. Whereas motor fatigability may influence perceptions of fatigue, fatigue and fatigability are independent constructs, and it is unclear whether individuals with heightened MS-related fatigue universally experience heightened fatigability (24). Our group (25) speculated that a deteriorated resistance to fatigability could result in MS-related fatigue accumulation in response to daily life activities and subsequently reduce functional capacity, leading to the accumulation of fatigue over time. In an effort to avoid fatigue accumulation, PwMS may use energy conservation strategies, such as reducing their level of physical activity during the day, which could increase deconditioning and, in turn, exacerbate fatigability in a vicious circle (26, 27).

Over the last three decades, research has examined the relationship between fatigability and fatigue in PwMS (24). However, the majority of studies have explored fatigability in PwMS by testing small muscle groups (28). The lack of investigation into the relationship between fatigability induced by dynamic, large muscle-mass tasks (that are more representative of tasks of daily living and exercise) and perceptions of fatigue hinders our understanding of the relationship between MS-related fatigue and fatigability (28, 29). It is also possible that both fatigue and fatigability share a similar etiology and could both be related to disability level in PwMS. The severity of fatigability is indeed correlated to disability level among PwMS (30, 31), but fatigue severity and disability levels may (17, 32) or may not (33, 34) be related. However, it has been suggested that fatigue occurs in conjunction with pathological disease processes such that more severe fatigue is associated with the progression of disability over time (11, 35). Thus, a better understanding of how the mechanisms of fatigue and disability influence fatigability is warranted.

In the present review, we will examine the links between MS-related disability and fatigue and alterations to neuromuscular function that may explain muscle weakness and fatigability in PwMS. Most of the reviews on fatigue or fatigability in MS have focused on either methodology (28), the differences between PwMS and healthy individuals (36) or the pathophysiological mechanisms of fatigue (37, 38). Loy et al. (24) recently conducted a meta-analysis on the relationship between fatigue and fatigability. However, they did not explore the mechanisms underpinning these symptoms or the potential interplay between them. The purposes of this review are to (i) clarify the relationship between fatigue/disability and neuromuscular performance in PwMS and (ii) review the corticospinal and muscular mechanisms of voluntary muscle contraction that are altered by MS, and how they may be further influenced by disability level or fatigue.

## Muscle weakness and motor fatigability

### The interplay of MS-related disability on muscle weakness and motor fatigability

#### Muscle weakness

Maximal voluntary contraction (MVC) torque is one of the main indicators of neuromuscular capacity commonly used to assess motor function in PwMS (28). Although muscle weakness in PwMS differs from individual to individual depending on disability level and the location of neurological impairment (6), numerous investigations have found a lower MVC torque in PwMS compared to healthy individuals for muscles such as the quadriceps (30, 39–41), hamstrings (41), tibialis anterior (30, 39–43), first dorsal interosseous (42, 44) and other hand muscles (31, 45). The majority of studies that found no difference between PwMS and healthy controls (46–49) assessed muscles of the hand, a muscle group less affected by the disease (12). Yet, we recently reported that PwMS of low disability level may also display similar muscle strength as healthy controls in bigger muscle groups such as the quadriceps (50). Asymmetry in muscle strength, VO_2_peak or cycling workload has been evident between the contralateral bilateral limbs (51). However, Proessl et al. (52) observed no association between leg strength asymmetry and fatigability induced by walking, perceived fatigue or RPE in PwMS. As the level of neurological impairment increases, muscle strength is more severely impacted. People with secondary progressive MS who generally have higher EDSS scores are weaker in both the upper and lower limbs than those with relapsing remitting multiple sclerosis (44) or with lower EDSS scores (30, 31).

Since MS primarily affects the central nervous system (CNS), most researchers have attributed the reduced maximal torque observed in larger muscle groups to an impaired capacity to recruit motor units (36). The measurement of maximal voluntary activation (VA), using the interpolated twitch technique (53), quantifies the capacity to maximally activate motor units voluntarily, and is expressed as a percentage value (i.e., the ratio between a twitch superimposed to an MVC and a resting twitch). The studies that observed a lower MVC in PwMS also observed an impaired VA (39, 44, 54, 55). One of the likely reasons for this result is a reduced maximal motor unit discharge rate (56). Axonal damage in upper (from the brain to the spinal cord) and lower motor neurons (from the spinal cord to the muscle) may contribute to compromised motor unit activation (57); however, the assessment of VA does not delineate where the impairment within the CNS occurs. PwMS with higher EDSS scores (2.8 vs. 2.0) exhibited slightly, but significantly, lower VA (96 vs. 99%) (58) and people with SPMS demonstrated a lower VA than patient with RRMS (85 vs. 93%) (44). Furthermore, the previously mentioned studies that did not observe differences in MVC or VA between PwMS and healthy individuals tested PwMS of low disability level (EDSS = 2.0–2.5) (47, 48, 50).

Although the bulk of the literature has focused on alterations within the CNS to explain muscle weakness in PwMS, a few studies have investigated the changes in muscle contractile properties that likely occur secondary to deconditioning. Sharma et al. (59) showed that peak twitch torque and compound muscle action potential (M-wave) induced by a single-pulse electrical stimulus, as well as tetanic force evoked by trains of stimuli on the tibialis anterior muscle were lower for PwMS (EDSS = 5.1) compared to healthy controls. However, this observation was not supported by other investigations in PwMS of lower disability level where peak twitch torque was similar between the two groups for hand muscles (48) (EDSS = 2.5) and knee extensors (50) (EDSS = 2.0 for fatigued; 1.8 for non-fatigued). The divergent findings are likely due to the level of deconditioning in the MS participants compared to the control participants. In line with this thought, Coates et al. (50) matched the activity level in PwMS and healthy individuals that may potentially explain the lack of difference between groups.

#### Motor fatigability

**Figure 1:**
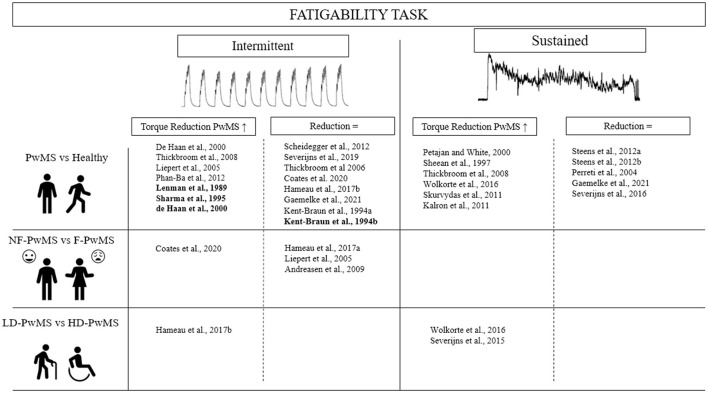
Comparison of fatigability between healthy subjects and patients with multiple sclerosis in function of fatigue and disability levels for isometric and intermittent fatiguing task. Bold, stimulated contraction; PwMS, Patient with multiple sclerosis; NF, Non-fatigued; F, Fatigued; LD, Low disability; HD, High disability.

Contrary to muscle weakness, fatigability induced by exercise has not been found to be consistently different in PwMS compared to healthy controls. This could be partially related to the large variety of exercise tasks employed (Figure 1). The decline in torque output during exercise (i.e., one of the main indices of fatigability) has been found to be higher in PwMS after tasks such as 3-min isometric MVCs of the abductor pollicis brevis and flexor carpi radialis muscles (60), a 45-s isometric MVC of the abductor pollicis (46), and a 2-min isometric MVC of the first dorsal interosseous (44) compared to healthy controls. Yet, some studies found no differences in fatigability between PwMS and healthy controls, particularly after contractions of the first dorsal interosseous (43, 47, 49) or abductor digiti minimi muscles (61). A caveat to most aforementioned studies is that they used hand muscles to test fatigability. In fact, these tasks are not representative of activities of daily living such as locomotion, where larger muscle groups and multi-joint movements are employed (62). However, small/distal muscles (e.g., of the hand) are affected later by the disease, allowing for people with higher disability levels to be included. In addition, because the exploration of the MS-related alterations in CNS mechanisms is more convenient in these muscles, they are often chosen for more fundamental studies. To circumvent this issue, some studies have compared exercise-induced fatigability in the lower limbs between PwMS and healthy controls. Skurvydas et al. (55) and Thickbroom et al. (42) observed significantly greater declines in torque output of PwMS when a 2-min MVC of the knee extensors and 15 s of intermittent dynamic contractions of the tibialis anterior were performed, respectively. Kalron et al. (63) similarly uncovered greater torque depression following 30 s MVCs of the knee extensors and flexors, as well as the ankle plantar flexors and dorsi-flexors in PwMS. Contrary to Skurvydas et al. (55), Gaemelke et al. (64) did not see heighted fatigability in PwMS following a 2-min MVC of the knee extensors, nor 40 maximal knee extensions. However, Gaemelke et al. (64) tested a cohort of PwMS of lower disability than the earlier study (EDSS = 2.4 vs. 3.5). Finally, greater fatigability in PwMS was not observed following 50 maximal isokinetic contractions of the knee extensors (41), nor after cycling to exhaustion (50). Functional tasks, such as the 6-min walk test, can be used to determine fatigability through the distance accomplished, or the change in walking speed from the beginning to the end of the test. Using this test, a greater fatigability was observed for PwMS compared to healthy controls (62, 63).

The discrepant findings may first be explained by the level of disability of the PwMS tested, as patients with higher EDSS scores have demonstrated higher fatigability following exercise tasks (28). For instance, Wolkorte et al. (44) found that people with SPMS (EDSS = 5.0) displayed a greater decline in torque during a 120-s maximal sustained finger abduction compared to people with RRMS (EDSS = 2.6). Severijns et al. (31) examined the change in handgrip strength after a 30-s maximal sustained contraction between PwMS of different disability levels and demonstrated greater fatigability in individuals with higher levels of disability (EDSS > 6 vs. EDSS < 6). A moderate but significant correlation (*p* = 0.35) between EDSS and fatigability was also observed in that study (31). The effect of disability on fatigability is also present during large muscle group, dynamic tasks whereby Hameau et al. (30) showed higher fatigability in PwMS with higher (EDSS = 5.0) vs. lower (EDSS = 3.5) disability scores after 50 isokinetic contractions of the quadriceps. Heightened fatigability is more likely to be present in PwMS with higher disability due to deteriorated integrity of the corticospinal tract and reduced functional motor connectivity that impairs VA (44, 65).

Furthermore, PwMS consistently displayed greater reductions in VA than healthy controls after sustained isometric contractions (45–124 s) of hand muscles (44, 46, 48, 55). Using functional magnetic resonance imaging to capture intracortical activity, it has been shown that PwMS have lower cortical activation during and following maximal fatiguing contractions, and unlike healthy individuals, they display an inability to increase cortical activity during the contraction, resulting in greater impairments in VA (47). PwMS also typically display higher compensatory activation of other brain regions compared to healthy controls during simple tasks (that are not necessarily intended to induce fatigability) such as 30-s of maximal finger-tapping (66–68). However, during or following fatiguing dynamic tasks, such as incremental cycling to exhaustion or 40 concentric contractions of the knee extensors, where fatigability was not heightened in the PwMS (mean EDSS: ~2.0–2.5), the reduction in VA was similarly not different between groups (50, 64). Dynamic tasks may also allow for more leeway for performance to be preserved (48). For example, although the absolute decline in knee extensor force was similar between PwMS and controls in a study by Hameau et al. (41), PwMS maintained a lower percent of their maximal torque through-out the 50 contractions, whereas the healthy controls produced a higher initial relative torque output that resulted in a sharper decline by the end of the contractions.

As central fatigue contributes to fatigability to a larger extent in PwMS, the exercise induced decline in muscle contractile function typically contributes less in PwMS compared to healthy individuals. Indeed, PwMS have demonstrated a lower reduction in force evoked by an electrical stimulus at rest (i.e., twitch force) following isometric contractions (45–124 s) of hand muscles (44, 46, 47) and in knee extensors (55). However, there has been some divergent findings (48). The previously mentioned dynamic exercise tasks that did not observe significantly greater central fatigability in the PwMS of low disability, consequently observed similar or greater peripheral fatigability in the PwMS (50, 64). The preservation of electrically evoked twitch forces in PwMS is due to a lower level of metabolic perturbation within the exercised muscles. Kent-Braun et al. (69) showed that decreases in PCr and increases in pH and Pi in response to intermittent isometric contractions of dorsiflexor muscles were lower in PwMS compared to healthy individuals. The lower level of peripheral disturbance is likely due to lower central motor activation (46, 48), thereby reducing metabolic demand within the muscle. In order to avoid the confounding effect of central limitations and further investigate the muscle contractile responses to exercise, four studies used tetanic stimulation to induce muscle fatigue in PwMS. These studies used different stimulation protocols: 180 × 240 ms at 50 Hz (total duration: 9 min) (59); 60 × 500 ms at 50 Hz (total duration: 90 s) at 30% of the MVC (39); 90 × 250 ms at 40 Hz (total duration: 3 min) at 20-50% of the MVC (70); and 180 × 240 ms at 50 Hz (total duration: 9 min) (71). All but one (71) of the studies found that force and the rate of force development declined to a greater extent in PwMS than healthy individuals during the electrically stimulated exercise of the quadriceps or tibialis anterior muscles (39, 59, 70). In two of the four studies, the recovery time was also longer for PwMS (59, 70) while it was not significantly different in the other two (39, 71). Moreover, a greater decrease in peak twitch was associated with a greater increase in half-relaxation time in PwMS than in controls and was attributed to a more severe impairment in muscle excitation-contraction coupling processes (59). Contrary to voluntary activation during exercise, the intensity of muscle contraction induced by electrical stimulation does not attenuate during the time course of stimulation protocols. Thus, it could be hypothesized that PwMS experience a lower metabolic stress and consequently a lower metabolite accumulation during constant load electrical stimuli than healthy controls (59, 71). This suggests that lower peripheral fatigue in PwMS during voluntary exercise could be due to compromised voluntary neural drive to the exercised muscle that prevents the development of muscle fatigue, while deconditioning likely promotes heightened peripheral fatigability in PwMS when the muscle is artificially fatigued.

In summary, the muscle weakness experienced by PwMS is primarily due to disease-related compromised neural drive (44, 55, 58, 72). During exercise, PwMS patients tend to display heightened fatigability compared to controls when the contractions are sustained and/or when disability status is higher and motor function is more likely to be impaired (31, 44, 73). When heightened fatigability is present in a task, VA is also more greatly impaired (46, 47, 74). As a result, available studies suggest that a concomitant preservation of muscle contractile ability exists for the PwMS (44, 46–48, 55). Indeed, electrically stimulated exercise results in greater force impairment in PwMS, reinforcing the hypothesis that impaired central neural drive preserves muscle function in PwMS compared to the healthy population (36).

### The interplay of MS-related fatigue on muscle weakness and motor fatigability

#### Muscle weakness

MVC torque has been found to be similar between PwMS experiencing higher vs. lower levels (i.e., sensation) of fatigue (highly fatigued = FSS score > 4 and/or MFIS score > 38 unless otherwise stated). This observation was consistent for both the upper (75, 76) and lower (30, 50, 58) limbs when EDSS was similar between groups. However, some studies found greater central contributions to muscle weakness in PwMS who experience fatigue. Andreasen et al. (58) showed that fatigued PwMS (FSS score >4) had a lower knee extensor VA than those who experienced less MS-related fatigue (FSS score < 4), but this observation was not supported by Coates et al. (50) who did not observe differences in knee extensor strength or VA in PwMS with higher vs. lower levels of MS related fatigue. In addition, the current literature suggests that the contributions of muscle contractile function to torque deficits in PwMS are not affected by the level of fatigue (50, 58).

#### Motor fatigability

Several theories have been formulated to explain the relationship between MS-related fatigue and fatigability (19, 77), but only a handful of studies have directly compared fatigability in PwMS experiencing higher vs. lower levels of fatigue for a similar level of disability (28, 30, 50). Andreasen et al. (58) found a similar decline in maximal quadriceps torque following short (4 s) isometric MVCs between fatigued and non-fatigued PwMS. Similarly, Hameau et al. (30) observed no difference between the two groups in quadriceps MVC torque decline after 50 maximal isokinetic contractions at 60°/s. However, following incremental cycling to task failure, the rate of decline in MVC torque was greater in fatigued PwMS compared to those with low levels of fatigue (50).

Similar to functional locomotor activity, cycling is a multi-joint task requiring repetitive activation of large muscle groups. During a functional task (i.e., the 6-min walk test), a higher perception of effort, along with an increase in self-reported fatigue, has been reported in PwMS compared to healthy controls (78, 79). Associated with higher perceived effort, the greater metabolic stress during dynamic exercise, such as cycling, may have resulted in greater decline in MVC torque observed in highly fatigued PwMS compared to the PwMS with low levels of perceived fatigue (80, 81). In line with this explanation, Taul-Madsen et al. (29) demonstrated that the rate of decline in maximal torque during dynamic contraction of lower limb muscles (i.e., 40 isokinetic knee extension contractions at 30°/s) was correlated to perceived fatigue severity, whereas a sustained isometric contraction was not. The incongruity in the current literature regarding the effect of MS-related fatigue on different measures of fatigability has also been highlighted in a meta-analysis by Loy et al. (24) who found a moderate relationship (*r* = 0.31) between the level of fatigue and fatigability in PwMS. This meta-analysis stated that the heterogeneity of included studies in terms of (i) applied exercise tasks and measures of fatigability (e.g., dynamic vs. isometric contractions for different durations of the exercises) (Figure 1), and (ii) the contribution of other confounding factors such as age, EDSS, MS phenotypes, and sex made it difficult to derive any conclusion about the relationship between MS-related fatigue and various indices of fatigability (e.g., declined MVC, VA, twitch force, time to task failure, etc.). Thus, further studies are required to properly understand the underpinning mechanisms that contribute to fatigability in PwMS with differing levels of fatigue. For example, the analysis of muscle electromyography and peripheral nerve stimulation during cycling at a similar relative intensity between fatigued and non-fatigued PwMS could allow us to understand how the neuromuscular system responds to the task being performed. This type of protocol has recently been used with cancer patients (82).

In the studies that investigated the central and peripheral contributions to fatigability, VA demonstrated similar rates of decline between high- vs. low-fatigued PwMS following an incremental cycling to exhaustion (50) and repetitive isometric MVCs (58), although more variability in VA was observed in the high-fatigue group during cycling (50). For peripheral fatigability, the decrease of potentiated twitch force was comparable in both groups when eight 4-s MVCs followed by a 15-s sustained isometric MVC of the quadriceps were performed (58). On the contrary, Coates et al. (50) found a greater reduction of knee extensors potentiated twitch force at exhaustion following dynamic whole-body exercise involving large muscle mass (i.e., cycling), suggesting that fatigued PwMS may show heightened peripheral fatigability during whole-body dynamic exercise. The authors attributed the result to the greater deconditioning in fatigued PwMS compared to the PwMS with low levels of fatigue.

To conclude, MVC torque and its associated central and peripheral neuromuscular determinants recorded at rest appear to be similar between fatigued and non-fatigued PwMS (30, 58, 72, 75). However, fatigability induced by dynamic exercise using larger muscle mass may be heightened in the more severely fatigued PwMS (30, 50). Due to the limited number of available studies, it is not possible to definitively explain the mechanisms underlying fatigability between fatigue and non-fatigued PwMS. However, it is possible that the study of corticospinal excitability may elucidate potential mechanisms that similarly underlie fatigability and MS-related fatigue in MS.

## Corticospinal responses

### The interplay of MS disability on corticospinal responses

#### At rest

As the corticospinal tract represents the primary pathway controlling voluntary movement, impairment in the integrity of this pathway could have important implications for impaired muscle force and functional capacity in PwMS (83, 84). In fact, lower MVC and reduced walking ability were correlated with brain corticospinal tract pathology among PwMS (85). One of the techniques to assess the integrity of the corticospinal pathway is transcranial magnetic stimulation (TMS). It can identify abnormalities in action potential transmission and excitatory and inhibitory processes within the CNS (86). This technique may characterize motor dysfunction beyond what is possible with traditional neuroimaging techniques such as magnetic resonance imaging (87–89). With this approach, an increase or decrease in the amplitude of the TMS-evoked short-latency excitatory response observed in the muscle electromyography, called the motor evoked potential (MEP), can be interpreted as a modulation of corticospinal excitability or a change in neuronal conduction (87, 90). A reduction in corticospinal excitability necessitates a higher cortical motor drive to maintain central activation. If excitability is reduced to an extent that it cannot be overcome with increased motor cortical input then motoneuron activation and force could be impaired (91). However, it should be noted that the mechanisms contributing to the modulation of MEP responses (e.g., the size of MEP amplitude, area, and silent period) are complex, and their relationship with voluntary activation and force production capacity is not fully understood (92).

The majority of studies have found smaller MEP amplitudes in PwMS compared to healthy controls in hand muscles (43, 76, 87, 93–100), and in the tibialis anterior (96, 101–103). Although in certain cases, MEP amplitude was the same as healthy controls (42, 46, 75, 104–106). Recently, lower MEP amplitudes and higher resting motor thresholds, were found for the weaker hand compared to stronger one among a large cohort of PwMS (*N* = 110). These differences were greater with higher disability level. Moreover, these impairments of the CNS were correlated to motor outcomes such as walking speed or dexterity (107). Smaller MEP amplitudes is often consistent with the slower conduction velocities that are associated with demyelination, as the more variable cortical input to the motoneuron pool leads to smaller MEP amplitudes and longer MEP durations (86, 107, 108). Smaller MEP amplitudes have been consistently associated with higher EDSS scores (87, 103, 109, 110), and more progressed disease subtypes (97, 99). Specifically, MEP amplitude is affected by pyramidal tract impairment, whereby PwMS with motor dysfunction display smaller MEP amplitudes than those with no impairment (100). Therefore, discrepancies in the literature may be due to the level of pyramidal tract impairment. Nonetheless, Kale et al. (87) found that 67% of PwMS with no pyramidal tract impairment also displayed smaller MEP amplitudes than healthy controls, potentially indicating that MEP amplitude may be able to detect subclinical pathologies (Table 1).

**Table 1:** Differences in TMS parameters between healthy controls and patients with multiple sclerosis in function of fatigue and disability level.

**Author**	**SUB GROUPS**	**Perceived Fatigue**	**EDSS**	**MS Type**	**Exercise**	**Target muscle(s)**	**Neuromuscular outcomes**
Liepert et al. (75)	MS-F: 8 MS NF: 8 HS: 6	MS-F FSS: 5.3 ± 0.4 (4.9-6.1) MS-NF FSS: 1.1 ± 0.2	MS-F: 3.1 ± 0.93 MS-NF: 2.9 ± 0.9	RRMS	Repeated contraction at 50% MVC until 50% MVC	SFD (Hand muscle)	↓ MEP-AMP MS-F=MS-NF=HS ↑ SICI MS-F>MS-NF
Perretti et al. (76)	MS-F: 32 MS-NF: 9 HS: 13	MS-F FSS: 51.6 ± 8.5 MS-NF FSS: 25.1 ± 11.8 HS FSS: 24.9 ± 6.4	MS-F: 3.4 ± 1. MS-NF: 2.3 ± 0.5	RRMS	Repeated contraction (30 s) at 50% MVC until 50% MVC	Thenar muscle (Hand muscle)	↓ MEP-AMP HS ↑ MEP-AMP MS-F=MS-NF
Russo et al. (111)	MS-F: 12 MS-NF: 12 HS: 10	MS-F FSS: 50.0 ± 7.0 MS-NF FSS: 20.0 ± 11.0	MS-F: 2.0 ± 1.0 MS-NF: 2.0 ± 1.0	RRMS	Repeated contractions during 5 min	Thumb muscle (Hand muscle)	↓ MEP-AMP MS-F↑ HS ↑ MEP PMF HS = MS-NF ↔ MEP PMF MS-F
Colombo et al. (112)	MS-F: 15 MS - NF: 15	MS-F FSS: 4.4 MS-NF FSS: 1.5	MS-F: 1.5 MS-NF: 1.5	RRMS	NONE	APB & AH (Hand muscle)	CMCT MS-F = MS-NF
Chalah et al. (113)	MS-F: 21 MS-NF: 17	MS-F FSS: 5.4 ± 1.0 MS-F MFIS: 32.8 ± 6.6	MS-F: 6.5 MS-NF: 6.0	RRMS: 6 PPMS: 16 SPMS: 16	NONE	FDI (Hand muscle)	SICI MS-F > MS-NF ICF MS-F = MS-NF SP MS-F = MS-NF
Coates et al., (50)	MS-F: 13 MS-NF: 13 HS:13	MS-F FSS: 5.4 ± 1.0 MS-F MFIS: 47.8 ± 10.5 MS-NF FSS: 2.5 ± 1.2 MS-NF MFIS: 21.6 ± 11.1 HS FSS: 1.8 ± 0.4 HS MFIS: 11.1 ± 10.7	MS-F: 2.0 ± 1.2 MS-NF: 1.8 ± 1.0	RRMS	Cycling beginning at a power output of 0.3 W/kg body mass and increasing 0.3 W/kg for stages 1–5 and 0.4 W/kg for further stages until volitional exhaustion	Lower limbs	↓ MVC MS-F > MS-NF VA MS-F = MS-NF ↓ PT MS-F > MS-NF ↔ MEP-AMP MS-F = MS-NF ↔ SP MS-F = MS-NF
Morgante et al. (95)	MS-F: 16 MS-NF: 17 HS: 12	MS-F FSS: 4.9 ± 0.8 MS-NF FSS: 2.2 ± 0.9	MS-F: 1.8 ± 0.6 MS-NF: 1.6 ± 0.6	RRMS	30 Reaction times	APB (Hand muscle)	RMT MS-F = MS-NF = HS AMT MS-F = MS-NF = HS CMCT MS-F &MS-NF > HS SICI MS-F = MS-NF = HS ICF MS-F = MS-NF = HS ↑ MEP-AMP HS & MS-NF > MS-F
Vucic et al (103)	MID: 25 MOD: 15 HS: 66	MID MFIS: 39.8 ± 3.6 MOD MFIS: 50.8 ± 3.3	MID: 1.6 ± 0.2 MOD: 5.9 ± 0.3	SPMS: 15 RRMS: 25	NONE	APB (Hand muscle)	SICI MOD > MID & HS MEP MOD > MID & HS ICF MOD > MID & HS RMT MOD > MID & HS CMCT MOD > MID > HS SP MOD = MID = HS
Tataroglu et al. (102)	MID: 37 MOD: 21 HS: 31	NA	MID: 1.7 ± 1.2 MOD: 4.5 ± 1.9	SPMS: 21 RRMS: 37	NONE	TA (Lower limbs)	MEP-AMP MOD & MID < HS CMCT MOD & MID > HS SP MOD & MID > HS
Conte et al. (97)	MID: 16 MOD: 14 HS: 17	NA	MID: 2.0 MOD: 6.0	RRMS: 16 SPMS: 14 HS	NONE	FDI (Hand muscle)	RMT MOD > MID & HS CMCT MOD > MID > HS MEP-AMP MOD < MID & HS SICI MOD > MID & HS
Facchetti et al. (114)	MID: 40 MOD: 13 HS: 20	NA	MID: 1.6 ± 1.1 MOD: 5.1 ± 1.3	RRMS: 40 SPMS: 13	NONE	TA (Lower limbs) ADM (Hand muscle)	CMCT MOD > MID > HS MEP-AMP MOD < MID
Petajan and White (60)	MS-NM: 16 MS-W: 16 HS: 10	NA	NA	NA	3-min hand-grip MVC	APB;FCR (Hand muscle)	CMCT MS-W = MS-NW = HS POST: ↑ CMCT MS-W & MS-NW > HS ↑ MEP MS-NM & HS > MS-W
Thickbroom et al. (42)	MS: 10 HS: 13	NA	MS: 2.0 ± 1.2	NA	5 series of intermittent tap with the foot (15-45s)	TA	MEP LAT MS > HS ↔ MEP-AMP MS = HS before exercise ↑ MEP-AMP MS > HS after
Thickbroom et al. (43)	MS: 23 HS: 15	MS-MFIS: 35.2 ± 17.2	MS: 2.3 ± 0.9	NA	120 isometric contractions (7-3s) at 40% of MVC	FDI (Hand muscle)	PRE: MEP-AMP MS < HS SP MS = HS DURING:↑ MEP-AMP MS > HS ↑ SP MS > HS POST: ↓ MEP-AMP MS > HS
White et al. (115)	MS: 11 HS: 11	MS-FIS: 61.0 ± 39.1 HS-FIS: 10.0 ± 9.2	MS: 1.9	NA	3-min MVC	APB (Hand muscle)	↔ MEP-LAT MS = HS ↔ CMCT MS = HS ↔ SP MS = HS
Sahota et al. (116)	MS: 30 HS: 30	NA	NA	RRMS SPMS	NONE	APB (Hand muscle) TA (lower limb)	CMCT MS > HS TA: MEP-AMP MS > HS APB: MEP-AMP MS = HS
Steens et al. (47)	MS: 20 HS: 20	MS: 5.3 ± 0.9 HS: 2.9 ± 0.6	MS: 2.5	RRMS	MVC isometric contraction during 124-s	FDI (Hand muscle)	CMCT MS > HS
Sheean et al. (46)	MS: 21 HS: 19	MS: 5.9 ± 0.9	MS: 5.4 ± 1.9	RRMS: 13 SPMS: 5 SPMS: 3	45-s isometric MVC	APB (Hand muscle)	RMT MS = HS CMCT MS > HS MEP-AMP MS > HS MEP LAT MS > HS ICF MS = HS POST: CMCT MS = HS MEP-AMP MS = HS
Di Sapio et al. (117)	MS: 28 HS: 28	NA	MS: 2.2	CIS: 2 RRMS: 17 SPMS: 2 PPMS: 3	NONE	Vastus medialis TA FHB	CMCT MS > HS MEP AREA MS < HS
Kale et al. (87)	MS: 131 HS: 53	NA	3 group: 0-2 2-4 >4	RRMS: 73 SPMS: 43 PPMS: 15	NONE	APB (Hand muscle)	MCT MS > HS MEP -AT MS > HS MEP-AMP MS < HS
Mills and Murray (118)	MS: 8 HS: 15	NA	NA	RRMS: 8	NONE	Forearm flexor muscles	MCT SA MS = HS MCT MCS MS > HS
Lenzi et al. (106)	MS: 18 HS: 18	NA	MS: 1.5	RRMS: 18	NONE	FDI (Hand muscle)	RMT MS = HS MEP-AMP MS = HS MEP-LAT MS > HS CMCT MS > HS
Schmierer et al. (119)	MS: 118 HS: 35	NA	EDSS: 4.9	RRMS: 96 PPMS: 19 SPMS: 3	NONE	FDI (Hand muscle) TA	FDI: MTH MS = HS FDI: CML MS > HS TA: CML MS > HS
Caramia et al. (104)	MS: 79 HS: 20	NA	REL-MS: 2.3 ± 0.7 REM-MS: 0.9 ± 0.8	RRMS: 79	NONE	Hand muscle	RMT REL-MS > REM-MS & HS SICI REL-MS = REM-MS & HS SP REL-MS < REM-MS & HS MEP-AMP REL-MS = REM-MS = HS
Schubert et al. (96)	MS: 11 HS: 10	FSS: 3.4-6.2	MS: 1.5-5	RRMS: 11	Walking (7-15min)	FHB (Hand muscle) TA	PRE: TA&FHB MEP MS < HS POST: TA&FHB MEP ↔ MS-HS TA&FHB CMCT ↔ MS-HS
Gagliardo et al. (101)	MS-D: 17 MS-ND: 15 HS: 20	NA	ND: 0-1.5 D: 2-3.5	RRMS: 32	NONE	TA	AMT MS-D > MS-ND RMT MS-D > MS-ND & MS-ND > HS MEP-AMP S-D > MS-ND & MS-ND > HS CMCT MS-D > MS-ND
Sahota et al. (116)	MS: 30 HS: 30	NA	NA	RRMS: 30	NONE	APB (Hand muscle) TA (lower limb)	RMT MS > HS CMCT MS > HS MEP-AMP MS < HS
Zeller et al (105)	MS: 22 HS: 22	NA	MS: 2.5	RRMS: 22	NONE	APB (Hand muscle)	MEP-AMP MS < HS
Bridoux et al. (98)	MS: 12 HS: 12	MS: 4.6 ± 0.4 HS: 2.1 ± 0.3	MS: 2.5 ± 1.4	RRMS: 11 SPMS: 1	6-min isometric at 25% of MVC	APB (Hand muscle)	PRE: MEP-AMP MS < HS POST: ↓ MEP-AMP MS & HS
Neva et al. (109)	MS: 26 HS: 11	NA	MS: 2.0	RRMS: 26	NONE	ECR (Hand muscle)	MEP DURATION MS > HS SP onset MS > HS RMT MS > HS MEP-LAT MS > HS
Mordillo-Mateos et al. (94)	MS: 17 HS: 16	MS: 4.7 ± 1.7 HS: 2.9 ± 0.9	MS: 5.1 ± 1.9	RRMS: 9 SPMS: 8	2-min hand-grip MVC	FDI (Hand muscle)	PRE: MEP-AMP MS < HS RMT MS > HS CMCT MS > HS POST: ↓ MEP-AMP HS
Bassi et al. (93)	MS: 18 HS: 18	MS: 2.1	MS: 1.0	RRMS: 18	30 blocks of 20 abductions of index finger	FDI (Hand muscle)	PRE: AMT MS = HS RMT MS > HS SICI MS < HS ICF MS = HS POST: ↑ MEP-AMP MS < HS ICF & RMT MS = HS ↓ SICI MS < HS
Cabib et al. (120)	MS: 20 HS: 13	NA	MS: 2.0	RRMS: 20	NONE	Wrist extensor	MEP-LAT MS > HS
Mohy et al. (121)	MS: 26 HS: 26	NA	NA	RRMS: 17 SPMS: 9	NONE	APB (Hand muscle) TA (lower limb)	APB: CMCT & MEP-AMP MS > HS TA: CMCT MS > HS & MEP-AMP MS < HS
Nantes et al. (99)	MS: 36 HS: 18	NA	RRMS: 2.1 ± 0.3 PPMS & SPMS: 4.2 ± 0.3	RRMS: 22 SPMS: 8 PPMS: 6	NONE	FDI (Hand muscle)	MEP-LAT PPMS > RRMS & HS SICI SPMS < HS, RRMS & PPMS MEP-AMP SPMS < HS & RRMS ICF SPMS = HS = RRMS
Nantes et al. (100)	MS: 43 HS: 29	NA	RRMS-I:13 RRMS-P: 30	RRMS: 43	NONE	FDI (Hand muscle)	RMT RRMS-I = RRMS-P = HS SICI RRMS-I = RRMS-P = HS MEP-AMP HS & RRMS-P > RRMS-I MEP-LAT HS & RRMS-P > RRMS-I

MS-F, Fatigued Multiple Sclerosis patients; MS-NF, Non-Fatigued Multiple Sclerosis patients; HS, Healthy subjects; PF, Primary fatigue; SF, Secondary fatigue; NF, Non-fatigued; FSS, Fatigue Severity Score; EDSS, Neurological disability score; RRMS, Relapsing-remitting Multiple sclerosis; SPMS, Secondary progressive multiple sclerosis; PPMS, Primary Progressive Multiple Sclerosis; MID, Mild disability; MOD, Moderate disability; SEV, Severe disability; MS-NM, Normal Multiple Sclerosis Patient; MS-W, Multiple Sclerosis patients with weakness; MVC, Maximal voluntary contraction; MEP, Motor evoked potentials; MEP-AMP, MEP Amplitude; MEP-LAT, MEP latency; SICI, Short intra-cortical inhibition; PMF, Pre-movement facilitation; AMT, Active motor threshold; APB, abductor pollicis brevis; AH, Abductor Hallucis; FDI, First dorsal interosseus; ADM, abductor digiti minimi; ECR, Extensor carpi radialis; TA, Tibialis anterior; FHB, Flexor hallucis brevis; SFD, Superficial Flexor Digitorum; MCT SA, Mean conduction time spinal cord to axilla; MCT MCS, Mean conduction time motor cortex to spinal cord; CML, Central motor latency; RRMS-I, RRMS with impaired dexterity function, RRMS-P, RRMS with preserved dexterity function; CMCT, Central motor conduction time; ICF, Intracortical Facilitation; SP, Silent Period; RMT, Resting motor threshold; REL-MS, Patient with multiple sclerosis in relapsing phase; REM-MS, Patient with multiple sclerosis in remitting phase. ↑, Increase; ↓, Decrease; → , No changes.

The assessment of the central motor conduction time (CMCT) and MEP latency (the duration between the stimulus and the onset of the MEP response) can be used to test the integrity of the central motor pathway in PwMS (86). The importance of these measurements resides in the fact that impairment in conduction velocity through the primary motor pathway has the potential to impact force production capacity (86). The CMCT is calculated by subtracting the latency of motor responses elicited by nerve stimulation at the level of the peripheral motoneuron from the latency elicited by magnetic stimulation at the motor cortex (122). PwMS have shown prolonged CMCT (46, 47, 97, 102, 116, 118) and a longer MEP latency compared to healthy controls (42, 76, 97, 99, 100, 102, 106, 109, 120). In addition, MEP latency was prolonged in PwMS with higher neurological impairments (87, 97), and more progressed disease subtypes (99). CMCT was also prolonged in PwMS with higher EDSS scores (5–9.5) compared to those with lower disability (0–4.5) (119, 121), and in People with secondary progressive MS compared to people with RRMS who had lower EDSS scores (97, 103, 114). Kandler et al. (88) highlighted that increased CMCT is more representative of pyramidal tract dysfunction than overall EDSS score as CMCT was correlated with motor disability but not EDSS score. CMCT is a measure of conduction velocity through the pyramidal tract, but the EDSS score is associated with both the pyramidal and non-pyramidal tract function (116).

During a muscle contraction, the MEP is followed by a period of muscle electromyography silence called the silent period (SP), which is reflective of corticospinal inhibition. The first 150 ms of the total SP duration is thought to be mediated by spinal responses, especially due to muscle spindle discharge, inhibition from Golgi tendon organ, activation of Renshaw cells (123) and activation of other inhibitory interneurons (124), and could contribute to the later part of the SP duration (125). The SP duration is also determined by altered activation of type B gamma-aminobutyric acid (GABA_B_) receptors (123–128). Moreover, it seems that the cortico-basal ganglia-thalamo-cortical loop could be involved in the SP modulation. Indirect and hyperdirect pathways could produce inhibitory projection to the thalamus and lead to the inactivation of the motor cortex (124). A perturbation in inhibitory circuitries at supraspinal or spinal levels could modulate the responsiveness of corticospinal network and potentially affect force production (129). Some studies reported a similar duration of SP between PwMS and healthy participants (43, 50, 113, 115), while other studies found a longer (100, 102), or shorter (130) SPs in PwMS. Longer SP was also displayed for the weaker hand compared to the stronger hand for PwMS, and this SP elongation was amplified with greater disability level (107). Vucic et al. (103) observed shorter SP durations in patients with SPMS compared to patient with RRMS, and Caramia et al. (104) found “relapsing” patient with RRMS to have reduced SP durations than healthy controls. The authors suggested that corticospinal hyper-excitability could occur due to an imbalance between glutamate and GABA that has been observed in the presence of acute neuronal damage (103, 104). On the other hand, upper limb motor dysfunction was associated with longer SP durations observed during remission in patient with RRMS, possibly because damaged interneuronal circuits could interfere with GABAergic activity and alter intracortical inhibition (100).

In addition to the single-pulse TMS measurements of MEP and SP, the paired-pulsed TMS paradigm allows for further exploration of intracortical inhibitory and facilitatory processes within the brain (122). Whereas the duration of the SP could be mediated by GABA_A_ and GABA_B_ receptors (126, 127), paired-pulse TMS with brief interstimulus intervals (e.g., 1–3 ms) can be used to quantify short-interval intracortical inhibition (SICI) mediated by the activity of GABA_A_ receptors (131, 132). Increasing the interstimulus interval to ~10 ms increases the MEP amplitude which further characterizes intracortical facilitation (ICF) (133). The activation of inhibitory and facilitatory interneurons mediating SICI and ICF, are thought to provide direct and indirect inputs into the corticospinal tract and play an important role in the regulation and fine-tuning of motor control (99, 109). The SICI measured on the superficial flexor digitorum and first dorsal interosseous was lower in PwMS than healthy participants (75, 93, 97, 104) but was the same in the first dorsal interosseous and abductor pollicis brevis muscles (95, 99, 100). For ICF, no differences were observed between healthy participants and PwMS (75, 95, 97, 104). However, People with secondary progressive MS (EDSS = 5.9–6.0) displayed a greater ICF and a lower SICI (i.e., greater cortical hyper-excitability) than people with relapsing-remitting MS (EDSS = 1.6–2.0) (97, 99, 103), and negative correlations (i.e., r = −0.71, Vucic et al. (103)) have also been found between SICI and EDSS score (97). As with SP, it is possible that intracortical inhibition is altered under acute disease related processes such as local inflammation or glutamate-mediated excitotoxicity, as reduced SICI was also present in people with relapsing remitting MS during relapse as compared to the remission phase (104, 134). This may also represent a compensatory neuro-plastic adaptation that occur to preserve motor-function (97, 103). The role of inflammatory process in corticospinal functioning was further highlighted, by Stamponi et al. (134), whereby SICI was increased and ICF reduced with anti-inflammatory cytokines. These anti-inflammatory cytokines could modulate the synaptic alterations in PwMS and limit the neuronal damage (134). However, a caveat to the current literature is that the lack of consistent findings in relation to corticomotor inhibition makes it difficult to determine whether altered intracortical facilitation and inhibition are significant contributors to force output deficits in PwMS.

In summary, TMS measures such as MEP amplitude, CMCT and MEP latency could be applied to detect demyelination or neuronal damage severity (within the pyramidal tract) in PwMS, in a severity-response relationship; however the correlation of these measures with central deficits and force production impairment is yet to be investigated (86). Indeed, perturbations to inhibitory and excitatory intracortical circuits and/or corticospinal excitability at rest seem to be more variable so that any conclusion could be derived regarding the association of these measures with motor dysfunction in PwMS (97, 103, 104).

#### During exercise

Lesion load and EDSS score are also correlated with the level of functional cortical reorganization (66, 135). PwMS with higher disability have a stronger activation of the ipsilateral motor and sensorimotor cortex (i.e., such as the ipsilateral inferior parietal lobule) for simple motor tasks (136) compared to PwMS with lower disability. Therefore, hyperactivity of secondary motor areas and spinal motoneurons seemingly occurs as an adaptive mechanism to compensate for disrupted neural pathways and connections. It may help preserve functional ability, but may be insufficient to maintain force output during maximal tasks (47). TMS studies have shown a greater increase in corticospinal excitability during intermittent exercise in PwMS, where larger increases in MEP amplitude were observed during 15-s of maximal foot tapping and 20-min of intermittent finger abduction in order to display similar task performance as healthy controls (42, 43, 135). However, Coates et al. (50) observed no increase in MEP amplitude during incremental cycling to exhaustion, so MEP amplitude remained consistently smaller throughout exercise in the PwMS compared to healthy individuals. In addition to parameters such as modality and intensity of exercise performed and muscle group tested, the level of motor disability in PwMS could be a plausible explanation for the divergent findings in the later three studies. Indeed, Coates et al. (50) recruited PwMS of lower disability compared to Thickbroom et al. (43) and Wolokorte et al. (42). Following exercise, lower (43), similar (46, 60, 75) and higher (76) MEP amplitudes have been reported in PwMS compared to healthy participants.

It is unclear whether the conduction of evoked potentials is affected by exercise and whether it contributes to heightened fatigability in PwMS. CMCT has been shown to be prolonged (60) or unchanged (115) following sustained isometric contractions of hand muscles where fatigability was heightened in the PwMS compared to healthy controls. Furthermore, Sheean et al. (46) and White et al. (115) found no difference in changes to MEP latency between PwMS and healthy individuals following sustained isometric exercise (45-s adductor pollicis MVC and 3-min maximal handgrip, respectively) whereby fatigability was greater in the PwMS. Similar results were found by Coates et al. (50) during brief knee extensor contractions measured intermittently throughout an incremental cycling task to exhaustion.

It is also unclear whether corticospinal inhibition is affected by exercise as Thickbroom et al. (43) observed elongation of the SP during submaximal intermittent isometric contractions of hand muscles in PwMS where fatigability was similar in both groups, but White et al. (115) observed a similar duration of SP between PwMS and healthy participants throughout fatiguing exercise whereby fatigability was heightened in the PwMS.

In summary, although the results are difficult to compare due to methodological differences between studies, functional cortical reorganization may occur in more highly disabled PwMS in order to maintain performance during simple motor tasks (135, 137), but it may be insufficient to maintain VA during fatiguing exercise (36, 44, 65). Unfortunately, the limited number of electrophysiological studies makes it difficult to discern how corticospinal excitability and inhibition are altered during exercise and how that may influence fatigability in PwMS.

### The interplay of MS-related fatigue on corticospinal responses

#### At rest

**Figure 2:**
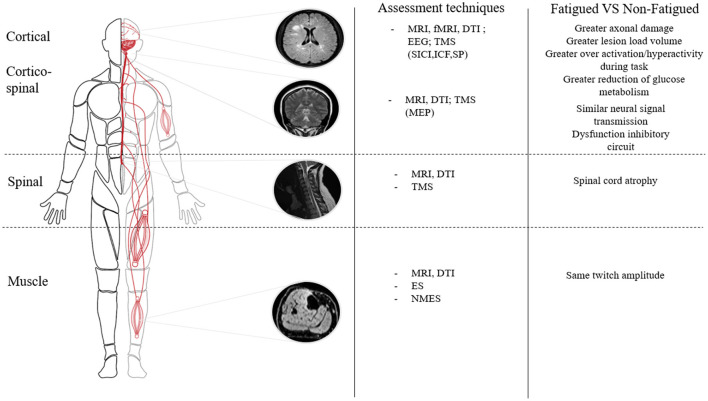
Differences in neurophysiological parameters within the neuromuscular system between fatigued and non-fatigued patients with multiple sclerosis. MRI, Magnetic Resonance Imagery; fMRI, functional Magnetic Resonance Imagery; DTI, Diffusion tension imaging; EEG, Electroencephalogram; TMS, Transcranial Magnetic Stimulation; SICI, Short Interval Cortical Inibition; ICF, Intracortical Facilitation; SP, Silent Period; NMES, Neuromuscular Electrical Stimulation; ES, Electrical Stimulation; MEP, Motor Evoked Potential.

Although diverse types of functional brain reorganization may be involved in fatigue in MS, brain regions involved in motor planning and execution are often implicated (138). During simple motor tasks or at rest, fatigued PwMS display an impairment of functional connectivity of the left sensory cortical network and frontal cortex compared to non-fatigued PwMS or healthy individuals (139, 140). An increased activation has also been demonstrated in fatigued PwMS compared to non-fatigued PwMS in secondary structures implicated in movement (e.g., the precuneus, cerebellum, and sensory motor cortex) when a decreased activation in the motor cortex and basal ganglia are present (141) (Figure 2). Alterations in basal ganglia functional connectivity (involved in the initiation and maintenance of movement) in fatigued PwMS were also demonstrated (142). Filipi et al. (143) uncovered an increased activation of the anterior cingulate cortex in fatigued compared to non-fatigued PwMS alongside reduced activation of other brain areas involved during movement (e.g., the ipsilateral cerebellar hemisphere and contralateral thalamus). These authors suggested that due to the higher cortical activation, fatigued PwMS may also have higher perceptions of effort which in turn could affect exercise performance. In fact, in a recent review, it was reported that many studies found that perceptions of effort increase more rapidly for PwMS compared to controls during fatiguing contractions (77). Although speculative, the repetitive cortical hyper-activation could potentially contribute to symptoms of fatigue induced by the accumulation of daily living tasks throughout the day. It is worth clarifying that in the aforementioned studies, fatigued and non-fatigued PwMS had similar and low EDSS scores (< 2) (139–141, 143). Since low levels of motor impairment exist at this low EDSS, it suggests that the over-activation of brain regions associated with motor tasks could have been involved in the pathophysiological mechanisms of MS-related fatigue, independent of disability level. Indeed, it was recently shown that fatigue severity was linked to altered basal ganglia functional connectivity, independent of disability level (142).

TMS measures of corticospinal excitability lend some support for the involvement of altered activation of the motor cortex and corticospinal tract in MS-related fatigue. MEP amplitudes recorded at baseline were the same in fatigued vs. non-fatigued PwMS for the upper limbs (75, 76), but MEP amplitudes in the knee extensors were smaller in highly fatigued PwMS when compared to healthy individuals (while MEP amplitude was not different from healthy individuals in PwMS with low fatigue) (50). In addition, corticospinal excitability was lower in fatigued than non-fatigued PwMS when measured immediately before a reaction time task. In this case, corticospinal excitability was inversely correlated to FSS score, displaying a pre-movement disfacilitation in those that experienced MS-related fatigue (95). According to (95), the MEP disfacilitation may reflect the involvement of brain areas implicated in motor planning more than a dysfunction in the transmission of the corticospinal drive, potentially indicating that corticospinal responses related to fatigue occur at the cortical level.

In line with Morgante et al. (95) hypothesis, it is possible that MS-related fatigue may be more related to altered pre-motor and motor-cortical activation than to impaired corticospinal transmission to the muscle (as is observed with heightened disability). Liepert et al. (75) observed no difference in MEP latency between fatigued and non-fatigued PwMS, and CMCT also appears to be similar between fatigued and non-fatigued PwMS for hand muscles (95, 112). However, both Coates et al. (50) and Perretti et al. (76) observed a longer MEP latency in highly fatigued PwMS compared to healthy individuals for lower and upper limbs, respectively. It has been suggested that MS-related fatigue may occur in conjunction with pathological disease processes such that more severe MS-related fatigue is associated with progression of disability over time (11, 35). Indeed, worsened disability over time, as shown by the changes of EDSS or brain atrophy, was linked to changes of MS-related fatigue questionnaires score. The disability or brain atrophy progression were the final consequence of the demyelination process while the functional brain reorganization occurred earlier to address the physical demands of life. This may explain some of the early signs of impaired corticomotor transmission that were present in the more highly fatigued PwMS (50).

Corticospinal inhibition has been correlated to fatigue severity (*r* = 0.34) measured *via* a visual analog scale in the first dorsal interosseus muscle (144), but no differences in baseline SP were present in fatigued vs. non-fatigued PwMS in the knee extensors (50). Similarly, intracortical inhibition measured *via* SICI on hand muscles was both greater (113), or the same (95) in fatigued and non-fatigued PwMS. Of note, studies that did observe heightened inhibition in the fatigued group involved PwMS with high EDSS (EDSS = 6–6.5 in (113) vs. 1.8 in (95)), so the role of disability cannot be disregarded. Liepert et al. (75) did observe an attenuation of SICI before exercise in hand muscles in fatigued PwMS that was not observed in non-fatigued PwMS or healthy individuals, demonstrating a lower pre-exercise inhibition in fatigued PwMS. However, participants in Liepert et al. (75) study had a higher EDSS than in Morgante et al. (95) work (3.1 vs. 1.8, respectively); thus, this observation corroborates the idea that the disability level could affect inhibitory processes. Non-invasive brain stimulation (e.g., repetitive transcranial magnetic stimulation, transcranial direct current stimulation) has recently been found to be a promising tool to reduce fatigue symptoms. It was observed that fatigue was improved for few weeks following stimulation, depending upon the stimulation site (145). Future research should utilize these techniques to evaluate whether subjective fatigue and fatigability can be improved in PwMS.

Overall, although altered cortical activation appears to be present during motor tasks in more highly fatigued PwMS, these findings have not been consistently supported by TMS measures of corticospinal excitability (75, 76, 112, 146). As muscle strength is not necessarily more greatly impaired in fatigued PwMS, it is unclear whether the brain functional reorganization affects force output, or whether it helps to preserve force output in the face of motor-cortical disruptions.

#### During exercise

The effects of MS-related fatigue on corticospinal responses and consequently on fatigability remain unclear. The change in MEP amplitude was similar for fatigued and non-fatigued PwMS following intermittent isometric hand-muscle exercise at 50% of MVC whereby fatigability was the same in both groups (75, 76). Similarly, no changes in MEP amplitude were identified during incremental dynamic whole-body exercise to exhaustion whatever the MS-related fatigue level (50). On the other hand, MEP duration increased throughout the incremental cycling in the highly fatigued PwMS only, suggesting that fatiguing exercise may exacerbate the disruption of action potential propagation that was observed at baseline in the highly fatigued group. Regarding corticospinal inhibition, the SP decreased more in the highly fatigued PwMS throughout cycling which may be indicative of reduced inhibition in the face of MS-related fatigue (50). Interestingly, intracortical inhibition was less in PwMS with higher cardiorespiratory fitness (144). Recently, the same team showed that the SP duration was reduced after 10 weeks of walking training in highly disabled PwMS (EDSS > 6)(147). Moreover, the decreased inhibition was associated with the reduction in fatigue measured by the FSS (rho = 0.76) and the MFIS (rho = 0.96). This promising result suggests that the beneficial effects of exercise on fatigue could be partially a result of neuroplasticity in the brain, even in highly disabled PwMS. However, further studies are needed to examine if this improvement in intracortical inhibition could improve functional capacity as well as fatigue.

In summary, although altered cortico-motor activation may be involved in MS-related fatigue, its influence on MS-related fatigability remains unclear (50, 72, 76). The diversity of the literature on corticospinal responses between PwMS and healthy individuals may be attributed to the different muscle groups tested, the MS subtype (e.g., RRMS vs. SPMS), the level of disability and the different methodological approaches used in the various studies. Due to paucity of research, it is difficult to provide a definitive statement on the impact of abnormal corticomotor function on motor performance in fatigued PwMS. Further studies are needed to clarify the central contribution to fatigability as a function of MS-related fatigue.

## Conclusion

The demyelinating and neurodegenerative processes involved in MS pathology affect the production of muscle torque and fatigability during exercise compared to the healthy population. The motor functional deficits observed in PwMS could be primarily attributed to the compromised central neural drive that occur to a greater extent with progressive MS-related disability and fatigue. This lower central command could also explain the reduced peripheral alterations observed in PwMS compared to healthy controls. Moreover, although the MVC torque at rest was similar regardless the fatigue level, fatigability was greater for patients with high compared to low level of fatigue. The impaired transmission of action potentials, as measured by CMCT, MEP latency and MEP amplitude, seems to contribute to muscle weakness in PwMS. However, its association with the heightened fatigability has yet to be determined. Alterations observed in the corticospinal excitability and inhibition of PwMS (e.g., increased MEP amplitude or decreased SP and SICI during exercise) may be indicative of compensatory activity utilized to preserve motor function in more highly disabled PwMS but these alterations have not been consistently observed in relation to muscle weakness or fatigability in PwMS. Cortical reorganization during motor tasks also seem to be a compensatory adaptation in patients with heightened MS-related fatigue, but there is no strong evidence that it explains muscle weakness or fatigability. However, the heightened cortical activation could influence perception of effort and in turn deteriorate motor performance in the more highly fatigued PwMS. Therefore, more studies on the relationship between fatigability and level of fatigue and disability need to be conducted on large muscle mass (e.g., quadriceps), ecological exercise (intermittent contractions, cycling etc…). Further investigation into the corticospinal responses of PwMS are also required.

## Author contributions

NR prepared figures and tables. All authors drafted the manuscript and edited and revised the manuscript, approved the final version of manuscript and agree to be accountable for all aspects of the work in ensuring that questions related to the accuracy and integrity of any part of the work are appropriately investigated and resolved. All authors designated as author qualify for authorship, and all those who qualify for authorship are listed. All authors conceived and designed the work.

## Conflict of interest

The authors declare that the research was conducted in the absence of any commercial or financial relationships that could be construed as a potential conflict of interest.

## Publisher's note

All claims expressed in this article are solely those of the authors and do not necessarily represent those of their affiliated organizations, or those of the publisher, the editors and the reviewers. Any product that may be evaluated in this article, or claim that may be made by its manufacturer, is not guaranteed or endorsed by the publisher.
